# Prognostic impact of clonal representation of myelodysplasia-related gene mutations in acute myeloid leukemia

**DOI:** 10.1038/s41375-025-02622-6

**Published:** 2025-04-28

**Authors:** Rabea Mecklenbrauck, Nora Borchert, Razif Gabdoulline, Piroska Poll, Carolin Funke, Maximilian Brandes, Louisa-Kristin Dallmann, Walter Fiedler, Jürgen Krauter, Arne Trummer, Bernd Hertenstein, Martin Müller, Michael Lübbert, Monika Schwalenberg, Andreas Voss, Nataliya Di Donato, Anke Bergmann, Verena Gaidzik, Konstanze Döhner, Hartmut Döhner, Arnold Ganser, Florian H. Heidel, Felicitas R. Thol, Michael Heuser

**Affiliations:** 1 Department of Hematology, Hemostasis, Oncology and Stem Cell Transplantation, Hannover Medical School, Hannover, Germany; 2 Molecular Haematology Unit, Weatherall Institute of Molecular Medicine, University of Oxford, Oxford, UK; 3 Department of Internal Medicine, University Hospital Hamburg Eppendorf, Hamburg, Germany; 4 Department of Hematology and Oncology, Municipal Hospital Braunschweig, Braunschweig, Germany; 5 Department of Hematology, Oncology and Palliative Care, Heidekreisklinikum, Walsrode, Germany; 6 Department of Internal Medicine I, Hospital Bremen-Mitte, Bremen, Germany; 7 Department of Hematology, Oncology and Immunology, Hospital Siloah, Hannover, Germany; 8 Department of Internal Medicine I, University Hospital Freiburg, Freiburg, Germany; 9 Department of Hematology, Oncology and Palliative Care, Hospital Lüdenscheid, Lüdenscheid, Germany; 10 Department of Oncology and Hematology, Klinikum Oldenburg, Oldenburg, Germany; 11 Institute of Human Genetics, Hannover Medical School, Hannover, Germany; 12 Department of Internal Medicine III, University Hospital Ulm, Ulm, Germany; 13 Leibniz Institute on Aging, Fritz-Lipmann Institute, Jena, Germany; 14 Department of Internal Medicine IV, University Hospital Halle (Saale), Martin-Luther-University Halle-Wittenberg, Halle, Germany

**Keywords:** Cancer genetics, Genetics research

## To the Editor

According to the international consensus classification (ICC) nine mutations define acute myeloid leukemia (AML) with myelodysplasia-related gene (MRG) mutations: *ASXL1, BCOR, EZH2, RUNX1, SF3B1, SRSF2, STAG2, U2AF1 and ZRSR2* [1]. Patients with MRG mutations have been shown to be older, leukopenic at diagnosis and overall have a worse outcome [2]. The 2022 European LeukemiaNet (ELN) classification assigns an adverse risk to patients with MRG mutations without any favorable genetic changes [3], who constitute up to 30% of AML patients [4–8].

Several studies have debated the prognostic impact of MRG mutations both with [9–11] and without favorable co-mutations [5–7, 12, 13] rendering heterogeneous results. As MRG mutations are acquired at an early stage of disease evolution and further mutations are acquired over the course of the disease [2], we hypothesized that considering clonality could provide a better understanding of the prognostic impact of MRG mutations.

Patients ≥ 18 years with newly diagnosed AML were included, who were diagnosed between 2000 and 2021 and underwent intensive induction treatment followed by either allogeneic hematopoietic cell transplantation (alloHCT) or consolidation chemotherapy. Patients from 15 academic centers in Germany were considered if molecular data from a myeloid panel, analyzed at Hannover Medical School, cytogenetic, and clinical data were available. At the time of diagnosis blood or bone marrow samples were analyzed via next generation sequencing using a 46 or 48 gene panel (Supplementary Table S1) as described before [14]. *ASXL1* c.1934 dupG was excluded if the variant allele frequency (VAF) was <18% based on internal validation. For mutations located on the X-chromosome the VAF was adjusted for hemizygosity in men.

Of the 550 cases identified, 153 patients (28%) were classified as ELN favorable risk, 158 (29%) as intermediate risk, and 239 (43%) as adverse risk (Supplementary Fig. S1).

Among ELN favorable patients, 31 (20%) had at least one MRG mutation. The mutational landscape is described in Supplementary Tables S2, S3.

Patients with MRG mutations within the ELN favorable risk group had similar baseline characteristics as favorable risk patients without MRG mutations (Supplementary Table S4). After a median follow-up of 5.4 years, patients in the favorable risk group had similar event-free (EFS) (HR 1.04, 95%CI 0.59–1.83, p = 0.88) and overall survival (OS) (HR 1.26, 95%CI 0.68–2.34, p = 0.46) with or without MRG mutations (Supplementary Fig. S2A, B). This also held true in multivariate analysis considering baseline risk factors and all mutations found in at least 10 patients (Supplementary Fig. S3).

A total of 255 MRG mutations were detected in 156 of the 239 patients in the ELN adverse risk group (Supplementary Tables S2, S3). Patients with MRG mutations showed a significantly worse EFS (HR 1.25, 95%CI 1.08–1.46, p = 0.003) and by trend OS (HR 1.19, 95%CI 1.0–1.42, p = 0.054) compared to ELN favorable risk patients (Supplementary Fig. S4A, B) but a similar EFS and OS compared to ELN intermediate risk patients (EFS: HR 1.19, 95%CI 0.9–1.59, p = 0.2; OS: HR 1.19, 95%CI 0.85–1.67, p = 0.3, Supplementary Fig. S4A, B). MRG mutated patients also displayed a similar EFS compared to the ELN adverse risk group (HR 0.84, 95%CI 0.61–1.16, p = 0.3), while OS was significantly longer compared to the ELN adverse risk group without MRG mutations (HR 0.61, 95%CI 0.43–0.87, p = 0.007; Supplementary Fig. S4A, B).

**Fig. 1:**
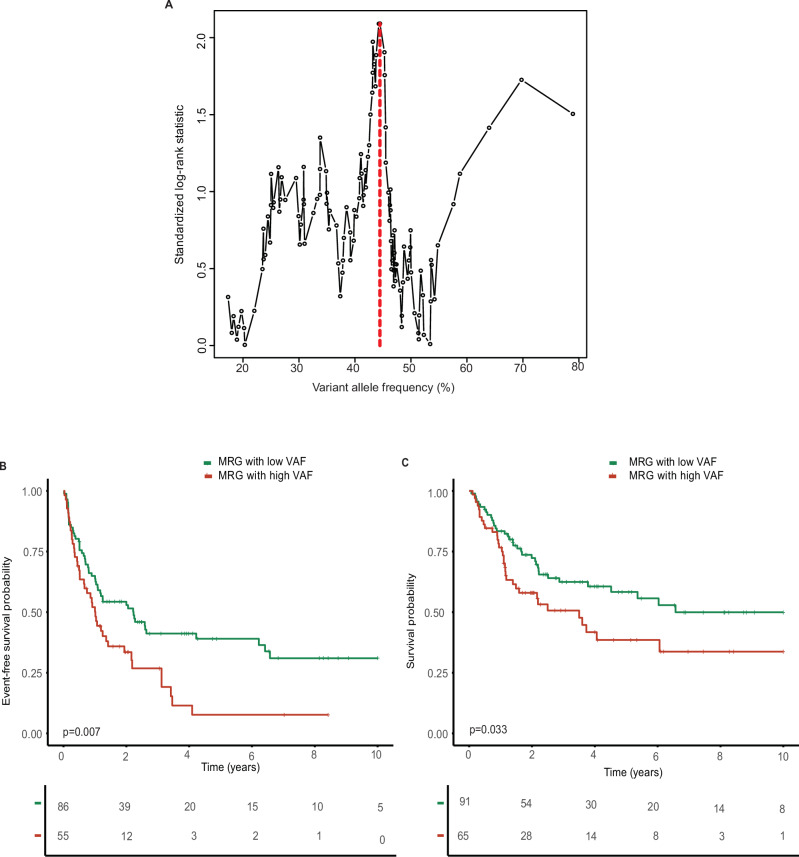
Prognostic impact of the size of the MRG mutated clone. **A** Among the 156 patients bearing at least one MRG mutation and classified as ELN adverse risk, the highest VAF was chosen to calculate a prognostically relevant VAF cut-off using maximally selected rank statistics. The most discriminating cut-off was 45%. **B** Event-free and **C** overall survival of patients with at least one MRG mutation with a VAF ≥ 45% compared to patients where all MRG mutations have a VAF < 45%.

Next, maximally selected rank statistics were used to determine the most prognostic threshold of the VAF in MRG mutations, which was found at 44.5% (Fig. 1A) [15]. This VAF cut-off was similar to the median VAF of all MRG mutations (41.9%). For simplicity we implemented a cut-off at a VAF of 45% for further analysis. First, the prognostic effect of a high or low VAF of MRG mutations was explored in the ELN favorable risk group. The 16 patients (10%) with low VAF and the 15 patients (10%) with high VAF had similar OS and EFS compared to the 122 ELN favorable risk patients without MRG mutation (Supplementary Fig. S5A, B).

Among the patients classified as ELN adverse risk, 91 (38%) patients in whom all MRG mutations had a VAF below 45% were classified as low VAF patients, while 65 (27%) patients with at least one MRG mutation with a VAF ≥45% were classified as high VAF patients. High VAF patients were significantly older and had a higher white blood cell (WBC) count and lower platelet counts at diagnosis (Supplementary Table S5). Notably, *ASXL1* was more likely mutated in the high VAF group (61%) whereas 76% and 66% of all *SF3B1* and *STAG2* mutations, respectively, were identified in low VAF patients (Supplementary Table S2). High VAF patients showed a significantly shorter EFS (HR 1.77, 95%CI 1.17–2.68, p = 0.007) and OS (HR 1.67, 95%CI 1.04–2.67, p = 0.033) as compared to low VAF patients (Fig. 1B, C).

Interestingly, low VAF patients had an EFS and OS which was comparable to ELN favorable and intermediate risk patients but a significantly better EFS and OS compared to adverse risk patients (low VAF vs. favorable – EFS: HR 1.13, 95%CI 0.95–1.34, p = 0.2; OS: HR 1.07, 95%CI 0.87–1.32, p = 0.5; low VAF vs. intermediate – EFS: HR 0.96, 95%CI 0.69–1.35, p = 0.8; OS: HR 0.96, 95%CI 0.64–1.44, p = 0.8; low VAF vs. adverse risk – EFS: HR 0.68, 95%CI 0.47–0.99, p = 0.043; OS: HR 0.49, 95%CI 0.32–0.75, p < 0.001) (Supplementary Fig. S6A–F). Conversely, high VAF patients showed a significantly worse EFS and OS than both ELN favorable and intermediate risk patients (high VAF vs. favorable—EFS: HR 1.52, 95%CI 1.26–1.84, p < 0.001; OS: HR 1.38, 95%CI 1.21–1.71, p = 0.003; high VAF vs. intermediate – EFS HR 1.76, 95%CI 1.22–2.54, p = 0.003; OS: HR 1.62, 96%CI 1.07–2.45, p = 0.023) whereas the outcome was comparable to ELN adverse risk patients (EFS: HR 1.19, 95%CI 0.80–1.78, p = 0.4; OS: HR 0.83, 95%CI 0.54–1.27, p = 0.4) (Supplementary Fig. S6A–F).

After data imputation, multivariate analysis of known risk factors in AML such as age, gender and WBC count at diagnosis as well as mutations found in at least 20 patients revealed that only the VAF of MRG mutations had a significant impact both on EFS and OS (Supplementary Fig. S7).

**Fig. 2:**
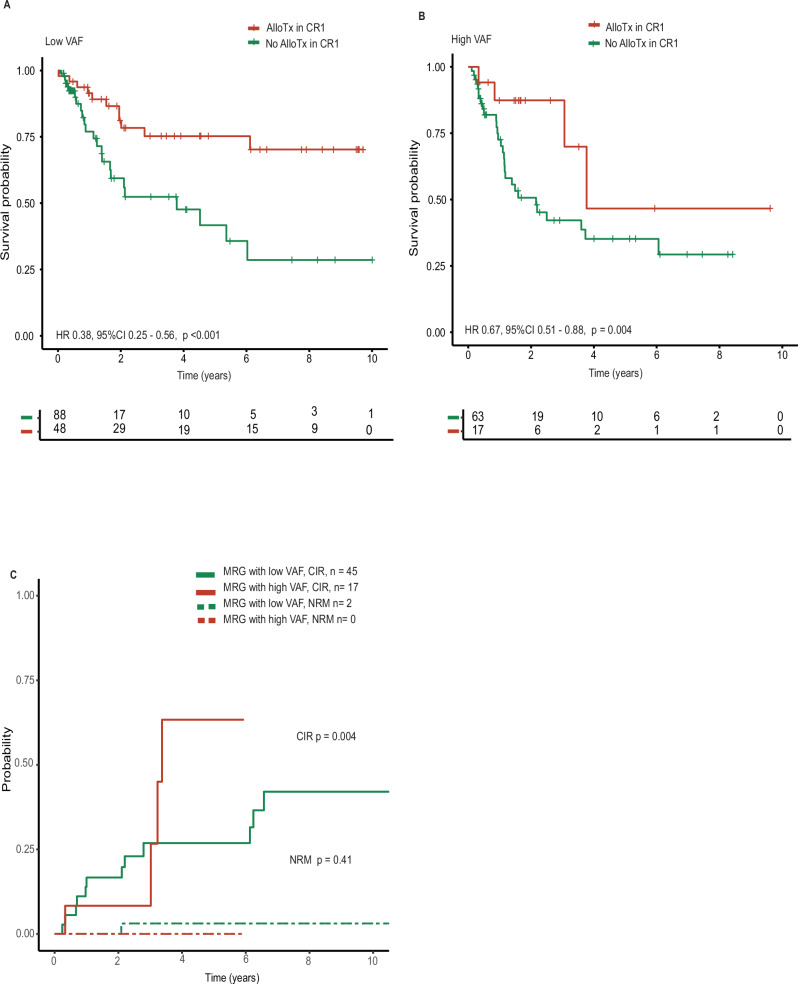
Impact of type of consolidation therapy on the outcome of patients with MRG mutations. **A** Simon-Makuch plot showing the time-adjusted overall survival of all low VAF patients considering allogeneic stem cell transplantation as a time dependent-variable. 3 patients had to be excluded from the analysis due to missing data. **B** Same as in A but for high VAF patients. 2 patients had to be excluded from the analysis due to missing data. **C** Competing risk analysis of MRG low vs. high VAF patients receiving alloHCT in first CR/CRi. CIR cumulative incidence of relapse, NRM non-relapse mortality. 3 patients had to be excluded from the analysis due to missing data.

For low VAF patients there was significant better OS when undergoing alloHCT in first complete remission (CR/CRi) (HR 0.38, 95%CI 0.25–0.56, p < 0.001). The same was seen for patients with a high VAF (HR 0.67, 95%CI 0.51–0.88, p = 0.004, Fig. 2A, B), albeit limited by small numbers. Finally, patients with a high VAF had a significantly higher cumulative incidence of relapse (CIR) compared to patients with low VAF MRG mutation (HR 3.48, 95%CI 1.48–8.16, p = 0.0042), while non relapse mortality and transplant associated characteristics were similar between these groups (HR 1.57, 95%CI 0.54–4.58, p = 0.41; Fig. 2C and Supplementary Table S6). This indicates that the more aggressive disease biology of MRG mutated AML with higher VAF is maintained even after alloHCT.

This study provides evidence that the clone size of MRG mutations is an independent prognostic marker in AML patients with MRG mutations.

The VAF cut-off of 45% separated the patients classified as ELN adverse risk into two distinct groups with different prognosis both in uni- and multivariate analysis. Patients with a VAF below 45% had an outcome similar to ELN intermediate risk patients, whereas patients who had at least one MRG mutation in the major clone with a VAF of 45% or higher had a significantly worse outcome. As the probability to reach CR was comparable between the two groups, the worse outcome is driven by a higher risk of relapse rather than primary resistance to induction chemotherapy.

The high VAF either represents patients with heterozygous mutations, which are present in nearly all leukemic clones, homozygous mutations, or mutations in combination with loss of heterozygosity. These findings support the hypothesis that MRG mutations drive the more aggressive behavior of leukemic cells, when they are part of the dominant clone. Future studies using whole exome and single cell sequencing should evaluate if the zygosity of MRG mutations and their role within the clonal hierarchy of AML - for which the VAF is used as a surrogate - can shed further light on the prognostic impact.

Contrastingly, MRG mutations had no prognostic impact in patients with favorable cytogenetic or molecular changes, including with high or low VAF, in line with previous studies. A recent study of patients with *NPM1* mutations suggested that the negative impact of MRG mutations was superseded by *NPM1*-measurable residual disease (MRD) after two cycles of intensive chemotherapy, emphasizing the value of MRD [10].

In the present study, a lower proportion of patients with high VAF underwent alloHCT. However, time-dependent survival analysis suggested that both groups derived benefit from undergoing alloHCT in first remission irrespective of VAF.

Taken together, our study suggests that the VAF of MRG mutations determines the prognosis of patients with these mutations. Our approach takes the clone size and thus the position of MRG mutations in the clonal evolution into account. Furthermore, our data supports the ELN recommendation to consider alloHCT for all patients bearing MRG mutations within the ELN adverse risk group.

## Supplementary information

Supplemental Appendix

## Data Availability

Individual patient data will not be made available to protect personal health information. De-identified mutational information will be shared upon reasonable request to the corresponding author.

## References

[1] Arber DA, Orazi A, Hasserjian RP, Borowitz MJ, Calvo KR, Kvasnicka HM, et al. International Consensus Classification of Myeloid Neoplasms and Acute Leukemias: integrating morphologic, clinical, and genomic data. Blood. 2022;140:1200–28. 35767897 10.1182/blood.2022015850 PMC9479031

[2] Lindsley RC, Mar BG, Mazzola E, Grauman PV, Shareef S, Allen SL, et al. Acute myeloid leukemia ontogeny is defined by distinct somatic mutations. Blood. 2015;125:1367–76. 25550361 10.1182/blood-2014-11-610543 PMC4342352

[3] Döhner H, Wei AH, Appelbaum FR, Craddock C, DiNardo CD, Dombret H, et al. Diagnosis and management of AML in adults: 2022 recommendations from an international expert panel on behalf of the ELN. Blood. 2022;140:1345–77. 35797463 10.1182/blood.2022016867

[4] Tazi Y, Arango-Ossa JE, Zhou Y, Bernard E, Thomas I, Gilkes A, et al. Unified classification and risk-stratification in Acute Myeloid Leukemia. Nat Comm. 2022;13:4622. 10.1038/s41467-022-32103-8 PMC9360033 35941135

[5] Lachowiez CA, Long N, Saultz J, Gandhi A, Newell LF, Hayes-Lattin B, et al. Comparison and validation of the 2022 European LeukemiaNet guidelines in acute myeloid leukemia. Blood Adv. 2023;7:1899–909. 36441905 10.1182/bloodadvances.2022009010 PMC10172873

[6] Tsai XC, Sun K, Lo M, Tien F, Kuo Y, Tseng M, et al. Poor prognostic implications of myelodysplasia-related mutations in both older and younger patients with de novo AML. Blood Cancer J. 2023;13:4. 36599822 10.1038/s41408-022-00774-7 PMC9813374

[7] Attardi E, Savi A, Borsellino B, Piciocchi A, Cipriani M, Ottone T, et al. Applicability of 2022 classifications of acute myeloid leukemia in the real-world setting. Blood Adv. 2023;7:5122–31. 37327116 10.1182/bloodadvances.2023010173 PMC10477447

[8] Huber S, Baer C, Hutter S, Dicker F, Meggendorfer M, Pohlkamp C, et al. AML classification in the year 2023: How to avoid a Babylonian confusion of languages. Leukemia. 2023;37:1413–20. 37120689 10.1038/s41375-023-01909-w PMC10317829

[9] Eckardt J, Bill M, Rausch C, Metzeler K, Spiekermann K, Stasik S, et al. Secondary-type mutations do not impact outcome in NPM1-mutated acute myeloid leukemia – implications for the European LeukemiaNet risk classification. Leukemia. 2023;37:2282–5. 37679502 10.1038/s41375-023-02016-6 PMC10624615

[10] Cocciardi S, Saadati M, Weiß N, Späth D, Kapp-Schwoerer S, Schneider I, et al. Impact of myelodysplasia-related and additional gene mutations in intensively treated patients with *NPM1*-mutated AML. HemaSphere. 2025;9:e70060. 39816531 10.1002/hem3.70060 PMC11733593

[11] Chan O, Al Ali N, Tashkandi H, Ellis A, Ball S, Grenet J, et al. Mutations highly specific for secondary AML are associated with poor outcomes in ELN favorable risk NPM1-mutated AML. Blood Adv. 2024;8:1075–83. 38170740 10.1182/bloodadvances.2023011173 PMC10907389

[12] Rausch C, Rothenberg-Thurley M, Dufour A, Schneider S, Gittinger H, Sauerland C, et al. Validation and refinement of the 2022 European LeukemiaNet genetic risk stratification of acute myeloid leukemia. Leukemia. 2023;37:1234–44. 37041198 10.1038/s41375-023-01884-2 PMC10244159

[13] Sargas C, Ayala R, Larráyoz MJ, Chillón MC, Rodriguez-Arboli E, Bilbao C, et al. Comparison of the 2022 and 2017 European LeukemiaNet risk classifications in a real-life cohort of the PETHEMA group. Blood Cancer J. 2023;13:77. 37173322 10.1038/s41408-023-00835-5 PMC10182047

[14] Thol F, Klesse S, Köhler L, Gabdoulline R, Kloos A, Liebich A, et al. Acute myeloid leukemia derived from lympho-myeloid clonal hematopoiesis. Leukemia. 2017;31:1286–95. 27881874 10.1038/leu.2016.345 PMC7610466

[15] Lausen B, Schumacher M. Maximally selected rank statistics. Biometrics. 1992;48:73–5.

