# Supplementary material for: Prognostic impact of clonal representation of myelodysplasia-related gene mutations in acute myeloid leukemia
Source: Leukemia. 2025 Apr 28;39(7):1773–7. doi: 10.1038/s41375-025-02622-6 (PMC12208903; doi:10.1038/s41375-025-02622-6)
Supplement: Supplementary file 1 — Supplemental Appendix [file 41375_2025_2622_MOESM1_ESM.docx]

Mecklenbrauck et al. Supplemental Appendix

**Supplemental Appendix to**

**Prognostic impact of clonal representation of myelodysplasia-related gene mutations in acute myeloid leukemia**

Mecklenbrauck et al.

**Supplemental Methods**

**Patients**

Patients aged 18 years or older with newly diagnosed AML as defined by the 2022 International Consensus Classification (ICC) (1), who were diagnosed between 2000 and 2021 and underwent intensive induction treatment followed by either allogeneic hematopoietic cell transplantation (alloHCT) or consolidation chemotherapy, were included in this study. In this retrospective cohort patients were included from 15 academic centers in Germany if molecular data from a myeloid panel analyzed at Hannover Medical School, cytogenetic, and clinical data were available (2-7). Since 2012 patients were registered in the AMLSG BiO-registry (8). Written informed consent was obtained according to the Declaration of Helsinki (9) and the study was approved by the local ethics review committee (ethical vote 936-2011 and 1187-2011).

**Molecular and cytogenetic analysis**

Bone marrow or peripheral blood samples were obtained at the time of diagnosis. DNA was isolated with the Allprep DNA/RNA purification kit (Qiagen, Hilden, Germany). DNA sequencing libraries were prepared using a custom TruSight and Nextera myeloid panel (Illumina, San Diego, CA, USA) with 46 or 48 genes associated with acute leukemia and sequenced on the Illumina HiSeq sequencer or the MiSeq sequencer (**Supplemental Table S1**). Combined genome coverage was 86 kilobases. The hg19 genome was used as a reference genome in a shortened version to keep only the bases of the selected genes padded with 100 kilo basepairs (10)**.**  Sequence alignment was done using Burrow-Wheeler Aligner, algorithm “mem” and “paired-end”. BaseRecalibrator and UnifiedGenotyper were used for quality control and variant calling. Single nucleotide variants were annotated using the BaseSpace Annotation Engine (Illumina Variant Studio version 3.0.12) as well as 2 custom databases derived from our myeloid panel sequencing and published variants (11, 12). Genomic positions with a read depth of less than 100 reads and detected variants with a VAF <5% were excluded. *ASXL1* c.1934 dupG was excluded if the VAF was <18%. For mutations located on the X-chromosome (*BCOR, BCORL1, KDM6A, PHF6, SMC1A, STAG2, ZRSR2*) the VAF was adjusted for hemizygosity in men.

Cytogenetic analysis was done locally by G- and R-banding analysis and described according to the International System for Human Cytogenetic Nomenclature (13)**.**

**Statistical analysis**

For all statistical analyses R version 4.3.0 (packages cmprsk, dplyr, epitools, ggplot2, ggsurvfit, gtsummary, magrittr, plyr, survival, survivalAnalysis, survminer, tidyverse) was used.

Continuous variables were analyzed via Wilcoxon-rank-sum test. The χ^2^-test or Fisher’s exact test were used for categorical variables. Complete remission (CR), complete remission with incomplete blood count recovery (CRi) and relapse were defined according to ELN 2022 criteria (14). Overall survival (OS) was calculated from diagnosis to death or last follow-up. Event-free survival (EFS) was calculated as the time from diagnosis to death, relapse, last follow-up or failure to reach CR after two cycles of chemotherapy, whichever occurred first. Patients for whom the date of response was missing were excluded for EFS analysis but included in the analysis of OS. The median follow-up for survival was calculated using the reverse Kaplan-Meier estimate (15).

Competing risk analysis was performed using the Fine-Gray model (16). Survival was estimated using the Kaplan-Meier method and differences were compared by the log-rank test and with Cox proportional hazard modeling. The Simon-Makuch method with clock-back correction was employed to assess the impact of alloHCT considering alloHCT as a time-dependent variable as described by Bernasconi et al. (17). For univariate models to evaluate the effect of alloHCT, Cox regression models with alloHCT as a time-dependent variable were applied. The maximally selected log rank test was used for calculating the optimal variant allele frequency (VAF) cutoff (18). To assign patients with more than one MRG mutation to the high or low VAF cutoff-group the gene with the highest VAF was considered.

A two-sided p-value of less than 0.05 was considered statistically significant. For multivariate analysis MRG mutations, age, gender, white blood cell counts and mutations with ≥20 cases (for MRG in ELN adverse) or ≥10 cases (for MRG in ELN favorable) were included. Data imputation was done using the mean (for continuous variables) and median (for categorial variables).

**Supplemental Tables**

**Supplemental Table S1. NGS gene panels at diagnosis.**

| **Gene** | **Exons covered by 46 gene panel** | **Exons covered by 48 gene panel** |
| --- | --- | --- |
| *ASXL1* | exon 12 | exon 12 |
| *ASXL2* | exon 11 | exon 11, 12 |
| *BCOR* | whole gene | whole gene |
| *BCORL1* | whole gene | whole gene |
| *BRAF* | exon 15 | exon 11, 15 |
| *CALR* | exon 9 | exon 9 |
| *CBL* | exon 9 | exon 8, 9 |
| *CEBPA* | whole gene | whole gene |
| *CSF3R* | exon 16 and 17 | exon 14 - 17 |
| *CSNK1A1* | exon 3 and 4 | exon 3 and 4 |
| *DDX41* | whole gene | whole gene |
| *DNMT3A* | whole gene, except exon 1 | whole gene, except exon 1 |
| *ETNK1* | exon 3 | exon 3 |
| *ETV6* | whole gene | whole gene |
| *EZH2* | whole gene, except exon 1 | whole gene, except exon 1 |
| *FLT3* | exon 16 and 20 | whole gene |
| *GATA2* | exon 2 - 6 | whole gene, except exon 1 |
| *IDH1* | exon 4 | exon 3 - 10 |
| *IDH2* | exon 4 | whole gene |
| *JAK2* | exon 12, 14 | exon 12, 14 |
| *KDM6A* | whole gene | whole gene |
| *KIT* | exon 10, 13, 17, 2, 8, 9 | exon 2, 8-11, 13, 17 |
| *KRAS* | exon 2 - 5 | exon 2 - 4 |
| *MPL* | exon 10 | exon 10 |
| *MYC* | exon 2 | exon 2 |
| *NF1* | whole gene | whole gene |
| *NPM1* | exon 11 | exon 11 |
| *NRAS* | exon 2 - 5 | exon 2 - 5 |
| *PHF6* | whole gene, except exon 1 | whole gene, except exon 1 |
| *PPM1D* | exon 1 (partial), 2 - 5, 6 (partial) | whole gene, except exon 1 |
| *PTPN11* | exon 13, exon 3 | exon 13, exon 3 |
| *RAD21* | whole gene, except exon 1 | whole gene, except exon 1 |
| *RUNX1* | whole gene, except exon 1 | whole gene, except exon 1 |
| *SETBP1* | whole gene | exon 4 |
| *SF3B1* | exon 14, 16 | exon 13 - 16 |
| *SMC1A* | whole gene | exon 2, 11, 16, 17 |
| *SMC3* | whole gene | exon 2, 11, 16, 17 |
| *SRSF2* | exon 1 | exon 1 |
| *STAG1* | whole gene, except exon 1 | whole gene, except exon 1 |
| *STAG2* | whole gene, except exon 1 and 2 | whole gene, except exon 1 and 2 |
| *TET2* | exon 3 - 11 | exon 3 - 11 |
| *TP53* | whole gene | whole gene, except exon 1 |
| *U2AF1* | exon 2, 6 | exon 2, 6 |
| *WT1* | exon 7, 9 | exon 7, 9 |
| *ZBTB7A* | exon 2, 3 (partial) | whole gene, except exon 1 |
| *ZRSR2* | whole gene | whole gene |

**Supplemental Table S2. Frequency of MRG mutations in the ELN favorable and adverse risk groups.**

| **Mutation** | **All patients (n = 208)** | **ELN favorable**  **MRG**  **mutations**  **(n = 31,**  **15%)** | **ELN adverse MRG**  **mutations**  **(n=156,**  **75%)** | **MRG low**  **VAF**  **(n = 91,**  **44%)** | **MRG high**  **VAF**  **(n = 65,**  **31** **%)** |
| --- | --- | --- | --- | --- | --- |
| ***ASXL1*** (n, %)  Median VAF (%)  VAF range (%) | 53 (28)  36.9  (10.1 – 100) | 3 (5)  30.04  (23 – 47.26) | 46 (29)  39.08  (10.01 –  100) | 18 (39)  27.77  (10.22 –  44.46) | 28 (61)  47.01  (10.01 –  100) |
| ***ASXL1 c.1934 dupG***  *(n, %)* | 16 (30) | 0 | 16 (35) | 5 (28) | 11 (39) |
| ***ASXL1 other*** *(n, %)* | 37 (70) | 3 (100) | 30 (65) | 13 (72) | 17 (61) |
| ***BCOR*** (n, %)  Median VAF (%)  VAF range (%) | 30 (16)  32.3  (5.97 – 53.18) | 5 (16)  41.76  (11.9 –  53.18) | 20 (13)  34.765  (5.965 –  50.64) | 9 (45)  24.830  (5.965 –  33.760) | 11 (55)  45.47  (26.25 –  50.64) |
| ***EZH2*** (n, %)  Median VAF (%)  VAF range (%) | 13 (7)  34.8  (11.9 – 97.14) | 1 (3) 14.11 | 12 (8)  32.52  (11.90 –  97.14 | 6 (50)  23.86  (11.90 –  40.25) | 6 (50)  86.62  (20.29 –  97.14) |
| ***RUNX1*** (n, %)  Median VAF (%)  VAF range (%) | 67 (32)  41.91  (10.1 – 100) | 2 (6)  45.66  (41.64 –  49.68) | 62 (40)  42.35  (10.01 –  100) | 30 (48)  30.39  (10.01 –  44.46) | 32 (52)  47.91  (13.14 –  100) |
| ***SF3B1*** (n, %)  Median VAF (%)  VAF range (%) | 21 (10)  26.49  (5.6 – 46.32) | 2 (6)  7.13  (6.58 –  7.68) | 17 (11)  33.27  (5.60 –  46.32) | 13 (76)  26.49  (5.60 –  41.18) | 4 (24)  44.28  (29.57 –  46.32) |
| ***SRSF2*** (n, %)  Median VAF (%)  VAF range (%) | 53 (25)  40.71  (5.88 – 100) | 3 (10)  46.24  (19.99 –  53.56) | 45 (29)  39.93  (5.88 –  100) | 23 (51)  30.33  (5.88 –  42.53) | 22 (49)  47.89  (27.23 –  100) |
| ***STAG2*** (n, %)  Median VAF (%)  VAF range (%) | 48 (23)  36.7  (10.71– 87.71) | 11 (35)  36.02  (10.71 –  87.71) | 32 (21)  37.2  (11.69 –  50) | 21 (66)  35.48  (11.69 –  43.43) | 11 (34)  45.56  (25.14 –  50) |
| ***U2AF1*** (n, %)  Median VAF (%)  VAF range (%) | 1. (6) 38.68   (12.58 –  58.75) | 1 (3)  43.3 | 11 (7) 38.17  (12.58 –  58.75) | 6 (55) 25.75  (12.58 –  43.78) | 5 (45) 41.86  (38.17 –  58.75) |
| ***ZRSR2*** (n, %)  Median VAF (%)  VAF range (%) | 22 (11)  14.47  (5.505 –  57.85) | 9 (29)  12.56  (7.085 –  48.215) | 10 (6)  45.547  (5.505 –  57.850) | 4 (40)  10.655  (5.505 –  17.990) | 6 (60)  48.52  (44.41 –  57.85) |

Abbreviations: ELN, European LeukemiaNet; MRG, myelodysplasia-related gene; VAF, variant allele frequency

**Supplemental Table S3.** **Co-mutations of patients with MRG mutations within ELN 2022 adverse and favorable risk groups.**

| **Mutation** | **Patients with MRG within ELN favorable (n = 31)** | **Patients with MRG within ELN adverse (n = 156)** |
| --- | --- | --- |
| ***ASXL2* (n, %)** | 1 (3) | 2 (1) |
| ***BCORL1* (n, %)** | 1 (3) | 14 (9) |
| ***CBL* (n, %)** | 0 (0) | 6 (4) |
| ***CSF3R* (n, %)** | 0 (0) | 2 (1) |
| ***CUX1* (n, %)** | 0 (0) | 3 (2) |
| ***DDX41* (n, %)** | 0 (0) | 7 (4) |
| ***DNMT3A* (n, %)** | 17 (55) | 41 (26) |
| ***ETNK1* (n, %)** | 0 (0) | 2 (1) |
| ***FLT3-ITD* (n, %)** | 0 (0) | 12 (8) |
| ***FLT3-TKD* (n, %)** | 1 (3) | 8 (5) |
| ***GATA2* (n, %)** | 3 (10) | 5 (3) |
| ***IDH1* (n, %)** | 5 (16) | 13 (8) |
| ***IDH2* (n, %)** | 3 (10) | 38 (24) |
| ***JAK2* (n, %)** | 0 (0) | 6 (4) |
| ***KMT2A* (n, %)** | 0 (0) | 1 (1) |
| ***KDM6A* (n, %)** | 0 (0) | 3 (2) |
| ***KIT* (n, %)** | 1 (3) | 4 (3) |
| ***KRAS* (n, %)** | 2 (6) | 11 (7) |
| ***MYC* (n, %)** | 1 (3) | 1 (1) |
| ***NF1* (n, %)** | 2 (6) | 4 (3) |
| ***NRAS* (n, %)** | 7 (23) | 19 (12) |
| ***PHF6* (n, %)** | 1 (3) | 8 (5) |
| ***PPM1D* (n, %)** | 0 (0) | 1 (1) |
| ***PTPN11* (n, %)** | 1 (3) | 3 (2) |
| ***RAD21* (n, %)** | 2 (6) | 7 (4) |
| ***SETBP1*(n, %)** | 2 (6) | 8 (5) |
| ***SMC3* (n, %)** | 1 (3) | 2 (1) |
| ***TET2* (n, %)** | 8 (26) | 23 (15) |
| ***WT1* (n, %)** | 3 (10) | 5 (3) |
| ***ZBTB7A* (n, %)** | 0 (0) | 1 (1) |

Abbreviations: ELN, European LeukemiaNet; MRG, myelodysplasia-related gene

**Supplemental Table S4: Baseline characteristics of patients classified as ELN favorable according to MRG mutation status.**

| **Characteristic** | **All patients (n = 153)** | **MRG**  **(n = 31)** | **No MRG**  **(n=122)** | **p** |
| --- | --- | --- | --- | --- |
| **Age at diagnosis** |  |  |  | 0.2 |
| **Median – years** | 52 | 55 | 51.2 |  |
| **Range – years** | 22 – 76 | 29 – 76 | 22 – 73.9 |  |
| **Patient sex** |  |  |  | 0.44 |
| **Male – no. (%)** | 81 (53) | 17 (55) | 55 (45) |  |
| **Female – no. (%)** | 72 (47) | 14 (45) | 67 (55) |  |
| **ECOG performance status at diagnosis** |  |  |  | 0.06 |
| **ECOG 0-1 – no. (%)** | 114 (75) | 18 (58) | 96 (79) |  |
| **ECOG ≥2 – no. (%)** | 19 (12) | 7 (23) | 12 (10) |  |
| **No information – no. (%)** | 20 (13) | 6 (19) | 14 (11) |  |
| **AML type** |  |  |  | 0.54 |
| **De novo – no. (%)** | 126 (82) | 20 (65) | 106 (87) |  |
| **Secondary^^[[1]](#footnote-1)^^ – no. (%)** | 20 (13) | 5 (16) | 15 (12) |  |
| **Therapy-related – no. (%)** | 1 (1) | 0 | 1 (1) |  |
| **No information – no. (%)** | 6 (4) | 6 (19) |  |  |
| **WBC count – 10^9^/L** |  |  |  | 0.08 |
| **Median** | 34.4 | 64.05 | 33.9 |  |
| **Range** | 1 – 303.9 | 1 – 303.9 | 1 – 163.4 |  |
| **No information – no. (%)** | 10 (7) | 3 (10) | 7 (6) |  |
| **Hemoglobin – g/dL** |  |  |  | 0.64 |
| **Median** | 8.9 | 8.6 | 9.0 |  |
| **Range** | 4.3 – 13.7 | 5.3 – 12.3 | 4.3 – 13.7 |  |
| **No information – no. (%)** | 12 (7) | 3 (10) | 9 (7) |  |
| **Platelet count – 10^9^/L** |  |  |  | 0.38 |
| **Median** | 56 | 56 | 56 |  |
| **Range** | 4 – 454 | 7 – 333 | 4 – 454 |  |
| **No information – no. (%)** | 11 (7) | 3 (10) | 8 (7) |  |
| **CR/CRi** |  |  |  | 0.25 |
| **Yes – no. (%)** | 139 (91) | 26 (84) | 113 (93) |  |
| **No – no (%)** | 14 (9) | 5 (16) | 9 (7) |  |
| **No information – no. (%)** | 0 (0) | 0 (0) | 0 (0) |  |
| **Allogeneic HCT** |  |  |  | 0.42 |
| **In CR1 – no. (%)** | 9 (6) | 3 (10) | 6 (5) |  |
| **In CR2 – no. (%)** | 44 (29) | 10 (32) | 34 (28) |  |
| **No HCT – no. (%)** | 34 (22) | 5 (16) | 29 (24) |  |
| **No information – no. (%)** | 0 | 0 | 0 |  |

Abbreviations: CR, complete remission; CRi, complete remission with incomplete hematological recovery; ECOG, Eastern Cooperative Oncology Group; HCT, hematopietic stem cell transplantation; MRG, myelodysplasia-related gene; WBC, white blood cell

Secondary AML as defined by previous medical history

**Supplemental Table S5. Baseline characteristics of MRG mutated patients with high and low VAF.**

Abbreviations: CR, complete remission; CRi, CR with incomplete hematologic recovery; NA, not applicable; WBC; white blood cell; ECOG, Eastern Cooperative Oncology Group

^1^ Secondary AML as defined by medical history.

| **Characteristic** | **All patients (n = 156)** | **Low VAF (n = 91)** | **High VAF (n = 65)** | **p (MRG low vs. high VAF)** |
| --- | --- | --- | --- | --- |
| **Age at diagnosis** |  |  |  | 0.042 |
| **Median - years** | 59 | 57.22 | 60 |  |
| **Range - years** | 18 – 75.85 | 21.07 – 75.85 | 18 – 74 |  |
| **Patient sex** |  |  |  | 0.491 |
| **Male – no. (%)** | 98 (63) | 55 (60) | 43 (66) |  |
| **Female – no. (%)** | 57 (36) | 36 (40) | 21 (32) |  |
| **No information – no. (%)** | 1 (1) | 0 (0) | 1 (2) |  |
| **ECOG performance status at diagnosis** |  |  |  | 0.153 |
| **ECOG 0-1 – no. (%)** | 107 (68) | 68 (75) | 39 (60) |  |
| **ECOG ≥2 – no. (%)** | 9 (6) | 3 (3) | 6 (9) |  |
| **No information – no. (%)** | 40 (26) | 20 (22) | 20 (31) |  |
| **AML type** |  |  |  |  |
| **De novo – no. (%)** | 76 (49) | 46 (51) | 30 (46) | 0.825 |
| **Secondary^1^ – no. (%)** | 69 (44) | 41 (45) | 28 (43) |  |
| **therapy-associated**  **– no. (%)** | 4 (3) | 3 (3) | 1 (2) |  |
| **No information – no. (%)** | 7 (4) | 1 (1) | 6 (9) |  |
| **Complex karyotype** |  |  |  | 1 |
| **Absent – no. (%)** | 103 (66) | 67 (74) | 36 (55) |  |
| **Present – no. (%)** | 8 (5) | 5 (5) | 3 (5) |  |
| **WBC count – 10^9^/L** |  |  |  | 0.001 |
| **Median** | 8.9 | 3.6 | 18.3 |  |
| **Range** | 0.5 – 600 | 0.58 – 206.1 | 0.5 - 600 |  |
| **No information – no. (%)** | 30 (19) | 21 (23) | 9 (14) |  |
| **Hemoglobin – g/dL** |  |  |  | 0.159 |
| **Median** | 9.0 | 9.3 | 8.9 |  |
| **Range** | 4.2 – 14.4 | 4.2 – 14.4 | 5.1 – 13.3 |  |
| **No information – no. (%)** | 30 (19) | 20 (22) | 10 (15) |  |
| **Platelet count – 10^9^/L** |  |  |  | 0.002 |
| **Median** | 59.5 | 69 | 38 |  |
| **Range** | 4 – 860 | 6 – 860 | 4 – 766 |  |
| **No information – no. (%)** | 30 (19) | 20 (22) | 10 (15) |  |
| **CR/CRi** |  |  |  | 0.106 |
| **Yes – no. (%)** | 113 (72) | 71 (78) | 42 (65) |  |
| **No – no. (%)** | 37 (24) | 17 (19) | 20 (31) |  |
| **No information – no. (%)** | 6 (4) | 3 (3) | 3 (4) |  |
| **Allogeneic HCT** |  |  |  | 0.001 |
| **In CR/CRi 1– no. (%)** | 65 (42) | 48 (53) | 17 (26) |  |
| **Not in CR/CRi 1– no. (%)** | 86 (55) | 40 (44) | 46 (71) |  |
| **No information – no. (%)** | 5 (3) | 3 (3) | 2 (3) |  |
| **No. MRG mutations** |  |  |  | <0.001 |
| **< 2 – no. (%)** | 72 (46) | 30 (33) | 42 (65) |  |
| **≥2 – no. (%)** | 84 (54) | 61 (67) | 23 (35) |  |

**Supplemental Table S6. Comparison of transplant-associated characteristics between MRG mutated patients with low vs high VAF.**

Abbreviations: GvHD, graft versus host disease; MAC, myeloablative conditioning; MMUD, matched unrelated donor; MRD, matched-related donor; MUD, matched unrelated donor; RIC, reduced-intensity conditioning; VAF, variant allele frequency.

| **Characteristic** | **All Patients (n = 65)** | **Low VAF (n = 48)** | **High VAF (n = 17)** | **p** |
| --- | --- | --- | --- | --- |
| **Donor sex** |  |  |  | 1 |
| **Male – no. (%)** | 42 (65) | 31 (65) | 11 (65) |  |
| **Female – no. (%)** | 17 (26) | 12 (25) | 5 (29) |  |
| **No information – no. (%)** | 6 (9) | 5 (10) | 1 (6) |  |
| **Donor match** |  |  |  | 0.58 |
| **MRD – no. (%)** | 10 (15) | 8 (17) | 2 (12) |  |
| **MUD – no. (%)** | 38 (59) | 28 (58) | 10 (59) |  |
| **MMUD – no. (%)** | 10 (15) | 6 (13) | 4 (24) |  |
| **No information – no. (%)** | 7 (11) | 6 (13) | 1 (6) |  |
| **CMV status** |  |  |  | 0.025 |
| **Negative/negative – no. (%)** | 19 (29) | 18 (38) | 1 (6) |  |
| **Other – no. (%)** | 41 (63) | 26 (54) | 15 (88) |  |
| **No information – no. (%)** | 5 (8) | 4 (8) | 1 (6) |  |
| **Conditioning regimen** |  |  |  | 1 |
| **MAC – no. (%)** | 17 (26) | 13 (27) | 4 (24) |  |
| **RIC – no. (%)** | 36 (55) | 27 (56) | 9 (53) |  |
| **No information – no. (%)** | 12 (18) | 8 (17) | 4 (24) |  |
| **Acute GvHD** |  |  |  | 0.65 |
| **Low (grade 1 and 2) – no. (%)** | 13 (20) | 8 (17) | 5 (29) |  |
| **High (grade 3 and 4) – no. (%)** | 8 (12) | 6 (13) | 2 (12) |  |
| **No GvHD – no. (%)** | 28 (43) | 21 (44) | 7 (41) |  |
| **No information – no. (%)** | 16 (25) | 13 (27) | 3 (18) |  |
| **Chronic GvHD** |  |  |  | 0.43 |
| **Limited – no. (%)** | 7 (11) | 4 (8) | 3 (18) |  |
| **Extensive – no. (%)** | 2 (3) | 2 (4) | 0 |  |
| **No GvHD – no. (%)** | 43 (66) | 32 (67) | 11 (65) |  |
| **No information – no.(%)** | 13 (20) | 10 (21) | 3 (18) |  |

**Supplemental Figures**

**Supplemental Figure S1**


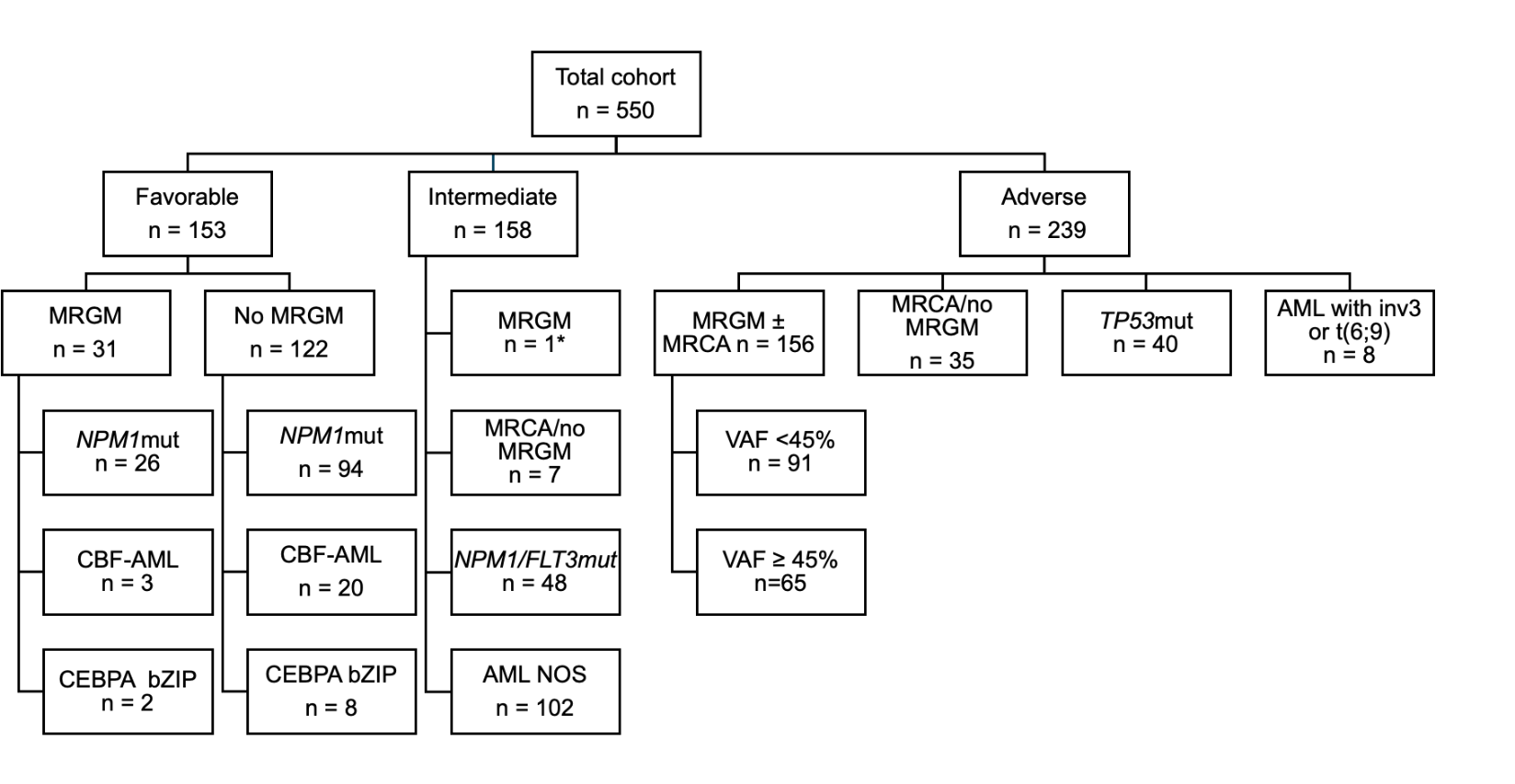


**Supplemental Figure S1. Consort diagram of the study cohort.**

* MRG mutation with concurrent t(9;11)

MRGM = myelodysplasia-associated gene mutation, CBF-AML = core binding factor AML, MRCA = myelodysplasia-associated cytogenetic abnormality

**Supplemental Figure S2**

**
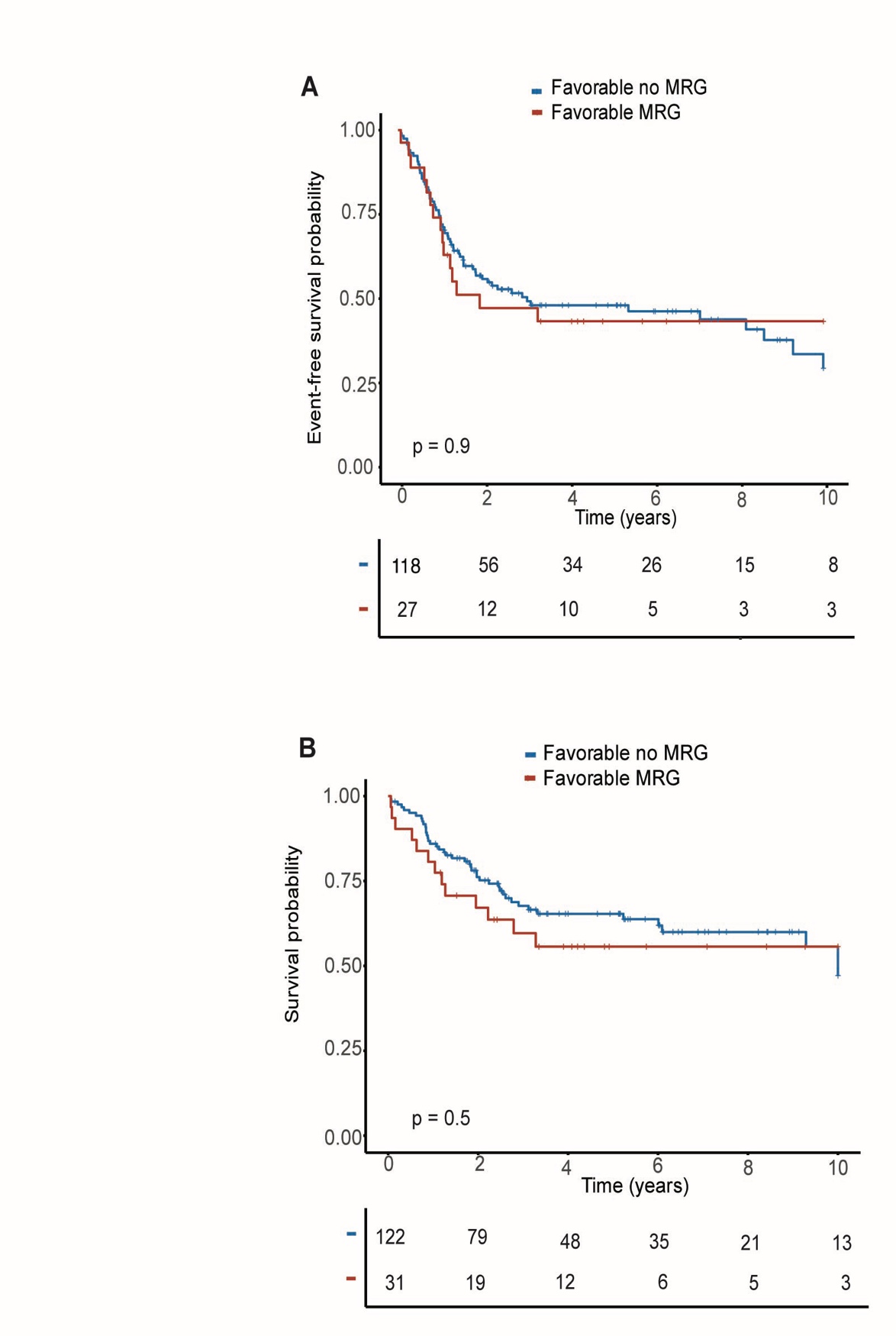
**

**Supplemental Figure S2: Outcome of AML patients in the ELN 2022 favorable risk group according to MRG mutation status.**

(A) Event-free and (B) overall survival of ELN 2022 favorable risk patients with and without MRG mutations.

**Supplemental Figure S3**


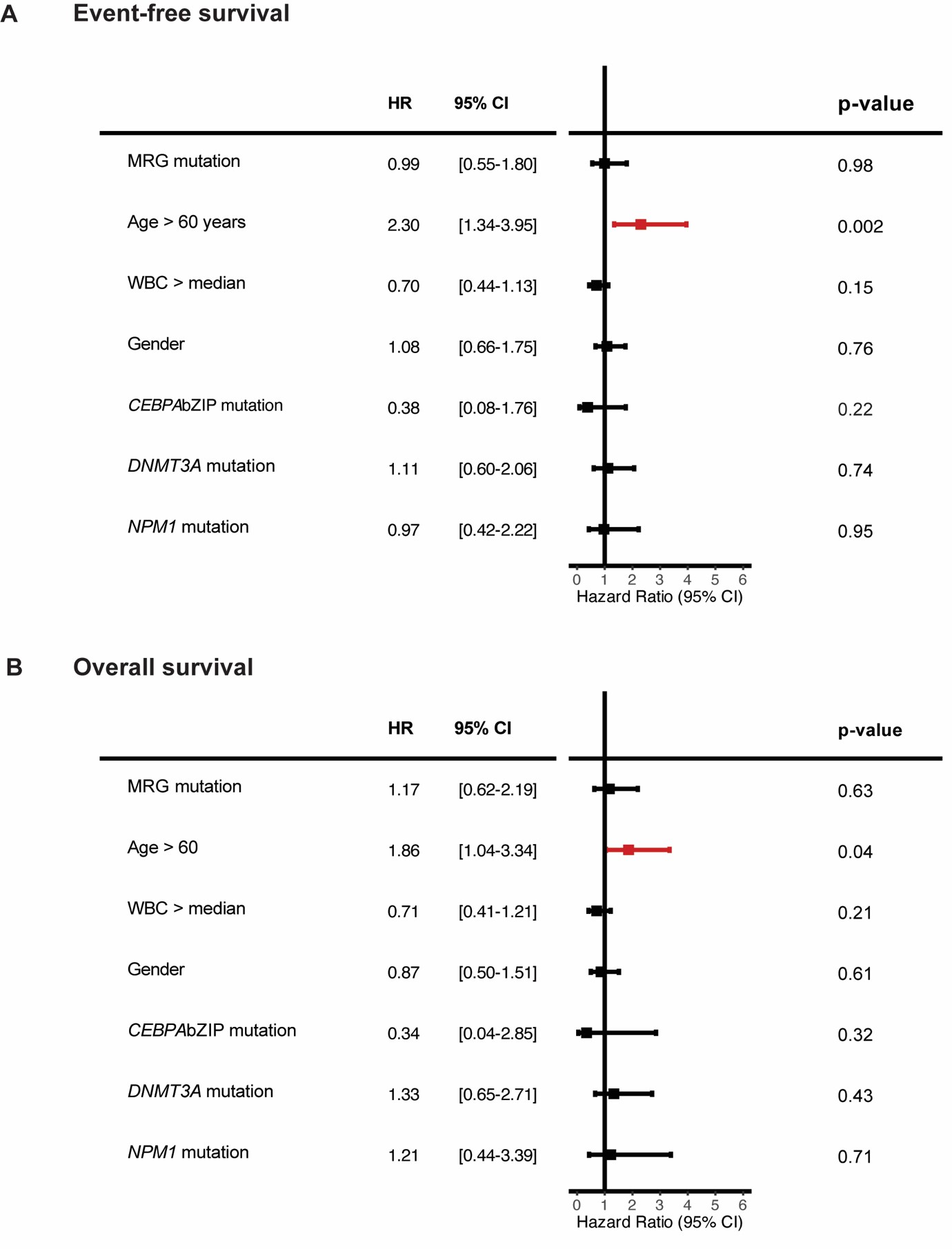


**Supplemental Figure S3:** **Multivariate analysis for (A) EFS and (B) OS in ELN favorable risk patients.**

Abbreviations: MRG, myelodysplasia-related gene; VAF, variant allele frequency

**Supplemental Figure S4**


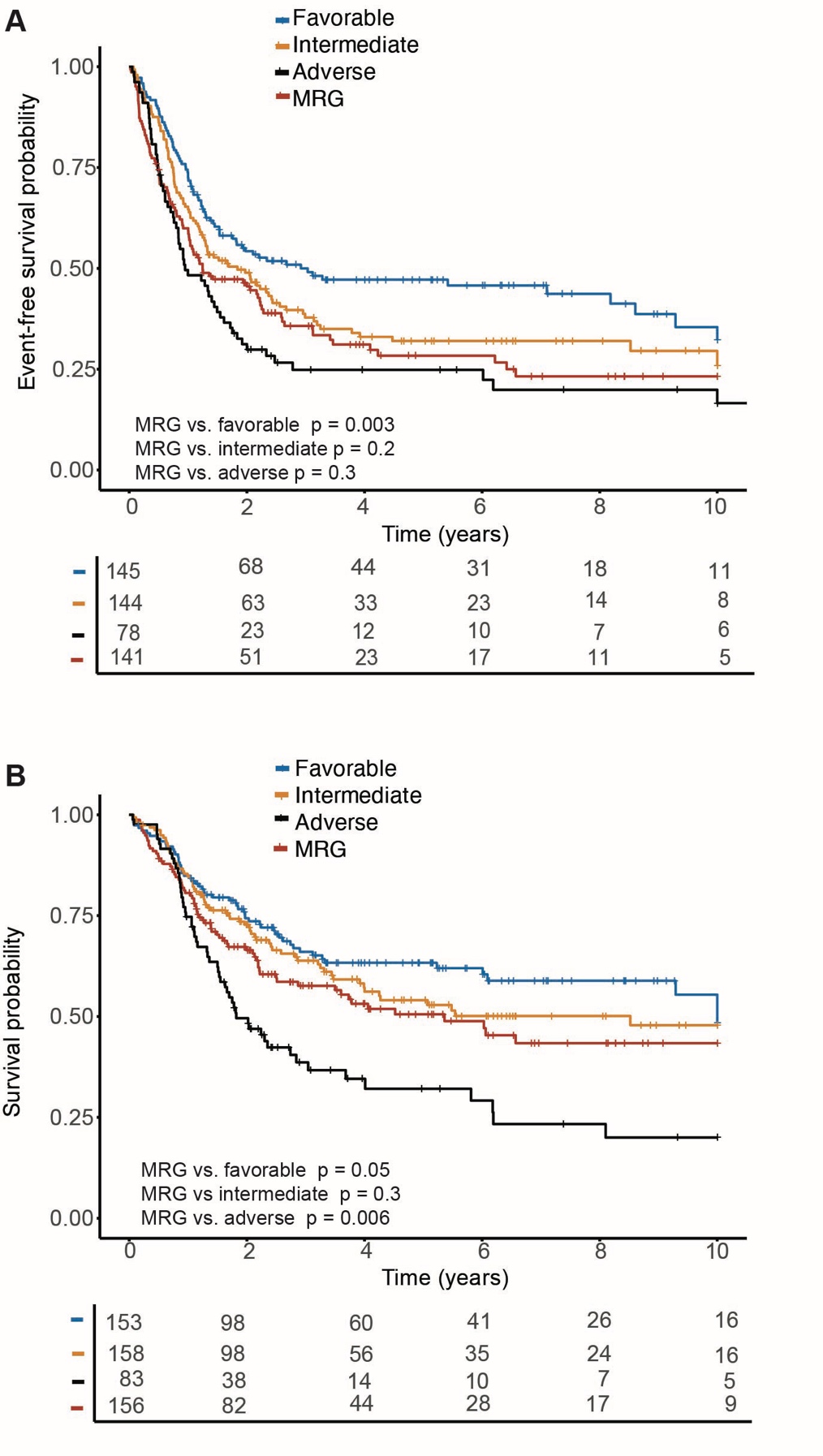


**Supplemental Figure S4: Outcome of patients with MRG mutations within the ELN 2022 adverse risk group compared to ELN 2022 favorable, intermediate and the remaining adverse risk groups.**

(A) Event-free and (B) overall survival of MRG mutated patients classified to the ELN adverse risk group in comparison to patients in the ELN 2022 favorable, intermediate and adverse risk groups.

**Supplemental Figure S5**

**Supplemental Figure S5: Outcome of MRG mutated patients within ELN 2022 favorable risk in comparison to ELN 2022 favorable w/o MRG mutations.**

1. Event-free survival of low and high VAF MRG mutated patients within ELN favorable risk in comparison to ELN favorable without MRG mutations.
2. Overall survival of low and high VAF MRG mutated patients within ELN favorable risk in comparison to ELN favorable without MRG mutations

**Supplemental Figure S6**


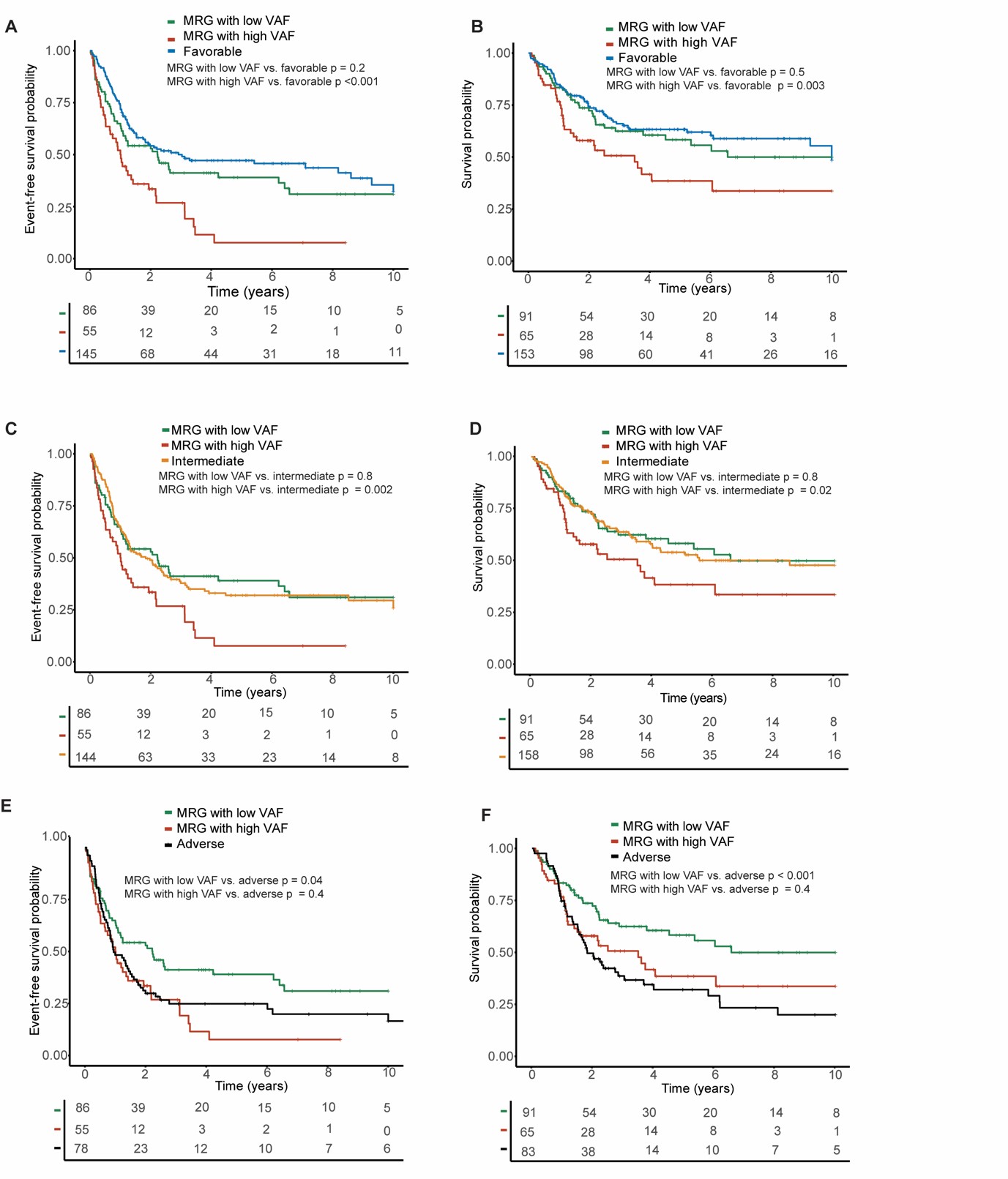


**Supplemental Figure S6: Outcome of MRG mutated patients within ELN 2022 adverse risk in comparison to ELN 2022 risk groups**

Event -free (left) and overall survival (right) of low and high VAF MRG mutated patients within ELN adverse risk in comparison to ELN favorable (A and B), ELN intermediate (C and D) and ELN adverse risk (E and F).

**Supplemental Figure S7**


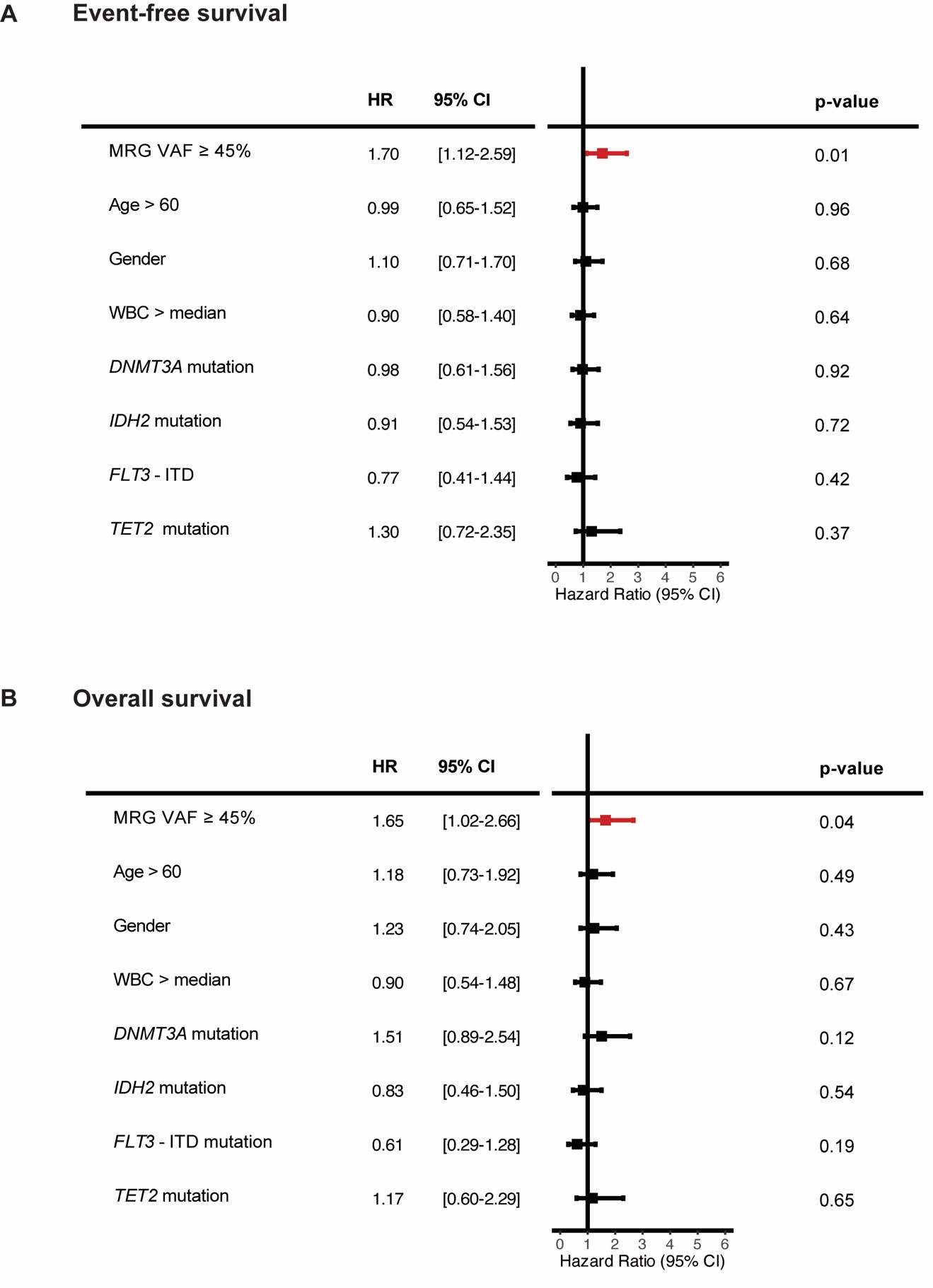


**Supplemental Figure S7.** **Multivariate analysis for (A) EFS and (B) OS in MRG mutated patients classified as ELN adverse risk considering a VAF ≥ 45% in at least one MRG mutation as potential adverse risk marker.**

Abbreviations: MRG, myelodysplasia-related gene; VAF, variant allele frequency

**References**

1. Arber DA, Orazi A, Hasserjian RP, Borowitz MJ, Calvo KR, Kvasnicka HM*, et al*. International Consensus Classification of Myeloid Neoplasms and Acute Leukemias: integrating morphologic, clinical, and genomic data. *Blood* 2022; **140**: 1200–28.

2. Stone RM, Mazzola E, Neuberg D, Allen SL, Pigneux A, Stuart RK*, et al*. Phase III Open-Label Randomized Study of Cytarabine in Combination With Amonafide L-Malate or Daunorubicin As Induction Therapy for Patients With Secondary Acute Myeloid Leukemia. *JCO* 2015; **33**: 1252–7.

3. Walker AR, Marcucci G, Yin J, Blum W, Stock W, Kohlschmidt J*, et al*. Phase 3 randomized trial of chemotherapy with or without oblimersen in older AML patients: CALGB 10201 (Alliance). *Blood Adv* 2021; **5**: 2775–87.

4. Larson RA, Mandrekar SJ, Huebner LJ, Sanford BL, Laumann K, Geyer S*, et al*. Midostaurin reduces relapse in FLT3-mutant acute myeloid leukemia: the Alliance CALGB 10603/RATIFY trial. *Leukemia* 2021; **35**: 2539–51.

5. Krauter J, Fiedler W, Schlenk RF, Paschka P, Thol F, Lübbert M*, et al*. Phase I/II study on cytarabine and idarubicin combined with escalating doses of clofarabine in newly diagnosed patients with acute myeloid leukaemia and high risk for induction failure (AMLSG 17‐10 CIARA trial). *Br J Haematol.* 2018; **183**: 235–41.

6. Schlenk RF, Döhner K, Krauter J, Gaidzik VI, Paschka P, Heuser M*, et al*. All-Trans Retinoic Acid Improves Outcome in Younger Adult Patients with Nucleophosmin-1 Mutated Acute Myeloid Leukemia – Results of the AMLSG 07-04 Randomized Treatment Trial. *Blood* 2011; **118**: 80.

7. Döhner H, Weber D, Krzykalla J, Fiedler W, Kühn MWM, Schroeder T*, et al*. Intensive chemotherapy with or without gemtuzumab ozogamicin in patients with NPM1-mutated acute myeloid leukaemia (AMLSG 09–09): a randomised, open-label, multicentre, phase 3 trial. *Lancet Haematol.* 2023; **10**: e495–509.

8. Nagel G, Weber D, Fromm E, Erhardt S, Lübbert M, Fiedler W*, et al*. Epidemiological, genetic, and clinical characterization by age of newly diagnosed acute myeloid leukemia based on an academic population-based registry study (AMLSG BiO). *Ann Hematol* 2017; **96**: 1993–2003.

9. World Medical Association: Declaration of Helsinki. Ethical principles for medical research involving human subjects [WMA web site]. 2024. https://www.wma.net/policies-posht/wma-declaration-of-helsinki/

10. Heuser M, Gabdoulline R, Löffeld P, Dobbernack V, Kreimeyer H, Pankratz M*, et al*. Individual outcome prediction for myelodysplastic syndrome (MDS) and secondary acute myeloid leukemia from MDS after allogeneic hematopoietic cell transplantation. *Ann Hematol* 2017; **96**: 1361–72.

11. Thol F, Klesse S, Köhler L, Gabdoulline R, Kloos A, Liebich A*, et al*. Acute myeloid leukemia derived from lympho-myeloid clonal hematopoiesis. *Leukemia* 2017; **31**: 1286–95.

12. Forbes SA, Bhamra G, Bamford S, Dawson E, Kok C, Clements J*, et al*. The Catalogue of Somatic Mutations in Cancer (COSMIC). *Curr Protoc Hum Genet.* 2008; **57**: 10.11.1,10.11.26.

13. McGowan-Jordan J, Hastings RJ, Moore S. ISCN 2020 : an international system for human cytogenomic nomenclature (2020).

14. Döhner H, Wei AH, Appelbaum FR, Craddock C, DiNardo CD, Dombret H*, et al*. Diagnosis and management of AML in adults: 2022 recommendations from an international expert panel on behalf of the ELN. *Blood* 2022; **140**: 1345–77.

15. Schemper M, Smith TL. A note on quantifying follow-up in studies of failure time. *Controlled Clinical Trials* 1996; **17**: 343–6.

16. Gray RJ. A Class of K-Sample Tests for Comparing the Cumulative Incidence of a Competing Risk. *Annals of statistics* 1988; **16**: 1141–54.

17. Bernasconi DP, Rebora P, Iacobelli S, Valsecchi MG, Antolini L. Survival probabilities with time-dependent treatment indicator: quantities and non-parametric estimators. *Statistics in Medicine* 2016; **35**: 1032–48.

18. Lausen B, Schumacher M. Maximally selected rank statistics. 1992. Biometrics **48,** 73-85

1. [↑](#footnote-ref-1)
